# Efficient Model for Coronary Artery Disease Diagnosis: A Comparative Study of Several Machine Learning Algorithms

**DOI:** 10.1155/2022/5359540

**Published:** 2022-10-18

**Authors:** Ali Garavand, Cirruse Salehnasab, Ali Behmanesh, Nasim Aslani, Amin Hassan Zadeh, Mustafa Ghaderzadeh

**Affiliations:** ^1^Department of Health Information Technology, School of Allied Medical Sciences, Lorestan University of Medical Sciences, Khorramabad, Iran; ^2^Department of Biostatistics and Epidemiology, School of Health, Social Determinants of Health Research Center, Yasuj University of Medical Sciences, Yasuj, Iran; ^3^Educational Development Center, Iran University of Medical Sciences, Tehran, Iran; ^4^Department of Risk Management, Smeal College of Business, Pennsylvania State University, State College, PA, USA; ^5^Department of Health Information Technology and Management, School of Allied Medical Sciences, Shahid Beheshti University of Medical Sciences, Tehran, Iran

## Abstract

**Background:**

In today's industrialized world, coronary artery disease (CAD) is one of the leading causes of death, and early detection and timely intervention can prevent many of its complications and eliminate or reduce the resulting mortality. Machine learning (ML) methods as one of the cutting-edge technologies can be used as a suitable solution in diagnosing this disease.

**Methods:**

In this study, different ML algorithms' performances were compared for their effectiveness in developing a model for early CAD diagnosis based on clinical examination features. This applied descriptive study was conducted on 303 records and overall 26 features, of which 26 were selected as the target features with the advice of several clinical experts. In order to provide a diagnostic model for CAD, we ran most of the most critical classification algorithms, including Multilayer Perceptron (MLP), Support Vector Machine (SVM), Logistic Regression (LR), J48, Random Forest (RF), K-Nearest Neighborhood (KNN), and Naive Bayes (NB). Seven different classification algorithms with 26 predictive features were tested to cover all feature space and reduce model error, and the most efficient algorithms were identified by comparison of the results.

**Results:**

Based on the compared performance metrics, SVM (AUC = 0.88, F-measure = 0.88, ROC = 0.85), and RF (AUC = 0.87, F-measure = 0.87, ROC = 0.91) were the most effective ML algorithms. Among the algorithms, the KNN algorithm had the lowest efficiency (AUC = 0.81, *F*-measure = 0.81, ROC = 0.77). In the diagnosis of coronary artery disease, machine learning algorithms have played an important role. Proposed ML models can provide practical, cost-effective, and valuable support to doctors in making decisions according to a good prediction. *Discussion*. It can become the basis for developing clinical decision support systems. SVM and RF algorithms had the highest efficiency and could diagnose CAD based on patient examination data. It is suggested that further studies be performed using these algorithms to diagnose coronary artery disease to obtain more accurate results.

## 1. Introduction

According to the World Health Organization (WHO), in 2020, cardiovascular disease, as one of the noncommunicable diseases, was the most important cause of death globally. Among cardiovascular diseases, coronary artery disease (CAD) is one of the most common diseases and the leading cause of death in developed countries. This disease is caused by the accumulation of platelets in the arteries or atherosclerosis, which blocks blood flow and increases the risk of heart attack and stroke. After the collection of fatty plaques and calcification, the blood vessels, which are initially soft and elastic, become narrow and hard, causing myocardial infarction in addition to narrowing the arteries [1–3].

Early CAD disease and early interventions to treat the disease and prevent its complications are vital strategies to combat and reduce mortality effectively. That is why healthcare providers use strategies to diagnose the disease early and start the treatment or intervention process early. In recent decades, numerous methods have been proposed for the early diagnosis of coronary artery disease. Many researchers across a wide range of scientific disciplines have argued that one of the most successful solutions for the early diagnosis of diseases, which has become increasingly common in recent years, is artificial intelligence (AI), which can improve diagnostic accuracy [3–6]. AI uses human intelligence to explore data relationships and mimic human problem-solving patterns. An essential category of AI is machine learning techniques that have played a pivotal role in diagnosing diseases. Machine learning (ML), as a common category of AI, has many applications in diagnosing and predicting different diseases. ML techniques have been used in various health research fields, and good results have been obtained. ML algorithms are a standard tool in knowledge exploration, which isused to create prediction and diagnosis models with high accuracy [7–9]. ML classification algorithms learn to analyze data by monitoring and discovering relationships between data. Monitoring by learning from labelled data allows us to identify data output that has never been seen before. This method is a general ML technique that gives a system a list of input-output pairs, and the system tries to find a function from input to output. This method is known as supervised learning since it requires some input data. However, there are specific issues for which supervised learning systems cannot generate the required output [10–15]. Previous studies on CAD datasets have included data from various care and diagnostic methods, including demographic information, lifestyle indicators, multiple examinations, patient laboratory test results, and cardiac tests such as ECG, exercise testing, etc. They had used the information. However, in the present study, only clinical examination data of patients and their demographic data were used to reach an early diagnosis. Therefore, the present study aimed to evaluate and compare different ML classification algorithms to determine an efficient algorithm for diagnosing coronary artery disease based on examination data of patients with coronary artery disease based on a clean dataset.

## 2. Related Work

With the increasing prevalence of CAD and the limitations of diagnostic tests, many studies have used several machine learning techniques to improve the accuracy of the adopted diagnostic methods.  Table 1 lists some relevant studies.

The studies were limited by the lack of clinical methods for selecting features. For these studies, two approaches were used to select features: either all features were used without data reduction techniques, or they were selected based on mathematical algorithms without considering their clinical relevance.

## 3. Methods

The present study aimed to design a model based on cheap clinical data for the first time in CAD diagnosis. In 2022, this study was carried out as a cross-sectional analysis of ML algorithms. To achieve the best performance in CAD data pattern recognition, different ML algorithms were used to analyze the data and compare their efficiency.

### 3.1. Dataset Description and Feature Selection

The used dataset is the Z-Alizadeh Sani dataset, which includes 303 data points from people suspected of having CAD, including 215 individuals who had CAD and 88 patients with normal which was their exact status confirmed using catheterization. This dataset contains 54 attributes (features) for every case in the dataset that can be utilized as CAD markers for patients (the features are arranged in four groups: demographics, symptoms and examination, ECG, and laboratory and echo features) This dataset is one of the most widely used datasets for automatic CAD detection in ML. A patient is diagnosed with CAD when one or more of his or her coronary arteries are stenosed. If the diameter of a coronary artery narrows by 50%, it is called stenosis [25].

In the present study, the development of a reliable selection model for diagnosing CAD disease was entirely based on clinical characteristics. These features were selected after consideration of the recommendations of three clinical cardiologists. A checklist was made to help determine the features. Following the registration of each feature using the checklist, the cost of measuring and registering each feature was calculated based on its accessibility. Finally, after this checklist was analyzed by the researchers, these clinical specialists checked and completed the checklists, and 26 features out of a total of 54 elements were chosen based on their clinical value and accessibility. By selecting these features from the original dataset, a dataset that was clinically useful and had features that had the most effects on the diagnosis of CAD was obtained. The models given in this research can be utilized to diagnose CAD using native data because access to the database with these features is more straightforward and less expensive (Table 2).

In the used dataset, the selected features were nominal (binary), and only age, weight, length, and BMI were numeric features. One of the strengths of the study is the use of cleaned data without any missing data. Utilization of this data has prevented the data preprocessing steps in the data mining process from being performed, and instead the data analysis process was performed directly using ML algorithms.

Data categorization is a crucial first step in using learning-based research models based on ML [15]. With no normalization and a ratio of 64 : 20 : 16, the dataset was divided into training, testing, and validation sets in the current study. Of the present research dataset, 80% was used for training (learning and validation), and 20% for testing.  Table 3 provides a detailed presentation of this classification. Additionally, to prevent the network from seeing data from a particular class during training, the training data were shuffled several times, each category comprising data with distinct labels.

In the present study, because the dataset is a standard public dataset and was collected under the supervision of a cardiologist, no preprocessing techniques including data cleansing and feature engineering were performed in this study.

### 3.2. Selective ML Algorithms

In light of the strengths of each ML algorithm and its capability to extract patterns from the datasets, several algorithms were used to find the most efficient algorithm for designing the diagnosis model of CAD. The algorithms selected include the multilayer perceptron (MLP), the support vector machine (SVM), the logistic regression (LR), the J48, the random forest (RF), the K-Nearest neighborhood (KNN), and the naive bayes (NB).

One of the most widely used ML algorithms is a neural network (NN), and the most prevalent algorithm on NN architectures is MLP which belongs to the class of supervised neural networks. An MLP network typically consists of three or more layers of nodes: an input layer that receives external inputs, one or more hidden layers, and an output layer that produces classification results. Errors are reduced using the gradient descent algorithm in this model [13, 26, 27].

SVM is one of the main supervised learning algorithms presented by Vladimir Vapnik within the area of statistical learning theory and structural risk minimization, which has been successfully applied to several classification and forecasting problems. The SVM has been applied to various problems related to pattern recognition and regression estimation, as well as medical diagnosis for disease classification. SVM is a powerful method for building classifiers. It allows the prediction of labels from one or more feature vectors by creating a decision boundary between two classes. Known as the hyperplane, this boundary is oriented to be as far away as possible from the closest data points from each class. The closest points are called support vectors [28].

(1) $$\begin{array}{c} \left(x_{1},y_{1}\right),\left(x_{1},y_{1}\right)\ldots \left(x_{n},y_{n}\right),x_{i}\in R^{d},y_{i}\in \left(-1,+1\right)\text{,} \end{array}$$

where *y*_*i*_ is the class label (positive or negative) of a training compound I and *x*_*i*_ is a feature vector representation. Thus, the optimal hyperplane is given by equations (1)–(3):

(2) $$\begin{array}{c} {Wx}^{T}+b=0. \end{array}$$

For all components of the training set, the *w* and *b* would meet the following inequalities:

(3) $$\begin{array}{c} Wx_{i}^{T}+b\geq +1y_{i}=1\text{,}\\ Wx_{i}^{T}+b\leq -1y_{i}=-1\text{.} \end{array}$$

**Figure 1:**
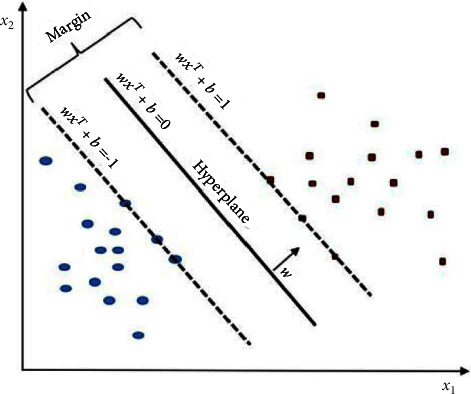
Linear SVM model (present a hyperplane to classify two classes).

Vectors *x*_*i*_ for which | *y*_*i*_ | *Wx*_*i*_^*T*^+*b*=1 will be termed support vectors (Figure 1).

LR is a type of nonlinear regression that takes categorical data as input. COX introduced the idea of LR in 1958, based on the principle of estimating a binary response based on a set of independent features. LR uses the logistic function to predict the probability of occurrence using the input feature set [29].

J48 is an upgraded version of the ID3 classification ML algorithm choice tree, which is based on a calculation called ID3 (Iterative Dichotomiser variant 3), developed by the WEKA undertaking group. The J48 calculation has a clear decision tree for the C4.5 gathering. There is a double tree in the situation. It is also known as a decision tree prediction algorithm for its steadiness in grouping issues. This way, simple, easy-to-understand rules can be constructed using this algorithm [30].

RF was introduced in 2001. The random space approach and bagging decision trees (DT) are the two methods that make up RF. The RF classifier comprises numerous DTs that have been trained using the bagging approach. After receiving the results of all DTs and voting on the results of all DTs, the final classification result is determined. Several classification and regression trees (CART) will be created by RF, each trained on a bootstrap sample of the original training data, and each searching a randomly chosen subset of input variables to find the split. By continually dividing the data in a node into child nodes, starting with the root node that holds the entire learning sample, binary decision trees known as CARTs are created. Each tree in the RF will vote for one or more inputs, and the majority vote of the trees will determine the classifier's output. High-dimensional data may be handled by RF, and the ensemble uses several trees. RF is a highly recommended classifier for dealing with situations like overfitting and underfitting. Noise and outliers can also be handled with RF. RF is a well-known classification technique that has been successfully applied to the categorization of a variety of medical datasets [31]. Some key characteristics of RF include:

1. It has a good method for guessing data that are absent.
2. Weighted random forest (WRF) is a technique for balancing inaccuracy in unbalanced data.
3. It calculates the significance of the classification's variables.

The simplest of all classifiers is KNN which belongs to the lazy learning algorithms family. Because KNN is a classifier-based instance, it may simply be constructed in parallel. In feature space, KNN utilizes majority voting among the labels for the K nearest data points, where K is an integer number. For continuous variables, Euclidean distance is used as a distance measure, while for discrete variables, hamming distance is used [15, 32] as a distance measure. The KNN classifier, also known as case-based reasoning, has been employed in a wide range of applications, including recognition and estimation. It is preferred over other classifiers due to its simplicity and high convergence speed [33, 34]. NB is a probabilistic statistical classifier with the advantages of accurate classification and excellent processing efficiency. When the input data have high dimensionality, NB is chosen. The label that optimizes the posterior probability is returned as an output in NB-based classification [35].

### 3.3. Metrics Evaluation

In this study, the performance of the selective classifier algorithms is evaluated via clinically meaningful statistical measures like precision, recall, F-measure, MCC (Matthews correlation coefficient), PRC (precision-recall curve) specificity, and F1 Score. To calculate these evaluation metrics, the following variables are required: TP (true positive), FP (false positive), which is how to calculate the indicators with the calculation formula.

The number of incorrect predictions of negative cases by the method. However, accuracy is not always a proper metric to evaluate model performance, especially in the case of an asymmetrical dataset. However, in this research, the accuracy metrics were to select the most efficient model through the selected pretrained networks. Equations (4)–(8) are used to briefly describe these measurements.

Accuracy: This parameter measures the ratio of accurately predicted cases to the total number of cases to assess a method's performance [15]. Mathematically, it is expressed as:

(4) $$\begin{array}{c} \text{Accuracy}=\frac{TN+TP}{TN+TP+FN+FP}\text{.} \end{array}$$

Recall, the ratio of observations in the actual classes that were correctly predicted as positive to all other observations [15, 32].

(5) $$\begin{array}{c} \text{Recall}\left(\text{sensitivity}\right)=\frac{TP}{TP+FN}\text{.} \end{array}$$

Precision, the ratio of observations in the actual classes that were correctly predicted as positive to all other observations [15, 32]. This metric shows how often different illness types are correctly classified.

(6) $$\begin{array}{c} \text{Specificity}=\frac{TN}{TN+FP}\text{.} \end{array}$$

F1 score, is one of the measures used frequently to assess a classifier's effectiveness. It is the harmonic mean of precision and recall [15]. The F-1 score is a relevant measurement for classification issues on unbalanced datasets, since it is more sensitive to data distribution.

(7) $$\begin{array}{c} F_{1}\text{ Score}=2\times \frac{\text{Precision}\times \text{Recall}}{\text{Precision}+\text{Recall}}\text{.} \end{array}$$

MCC (Matthews correlation coefficient), the most important metric that has been selected as the elective metric in the USFDA-led initiative MAQCII which aims at developing and validating predictive models for personalized medicine. The MCC is calculated as (5).

(8) $$\begin{array}{c} \text{MCC}=\frac{TP*TN-FP*FN}{\sqrt{\left(TP+FN\right)\left(TP+FN\right)\left(TN+FP\right)\left(TN+FN\right)}}\text{.} \end{array}$$

## 4. Result

In this research to reach high performance in the diagnosis of CAD using a clinical dataset, we selected seven different well-known classifiers for the diagnosis of coronary artery disease, based on the most frequently used ML algorithms in the field of diagnosis and classification of diseases. We assessed the efficacy of selective algorithms concerning patient data suspected of having CAD. The values for the performance metrics of the ML algorithms are shown in Table 3. In this table, eight proprietary metrics are used to assess the performance of ML algorithms. Based on the TP, PT, recall, F-measure, and MCC metrics for all algorithms implemented, it can be concluded that the highest amount of these metrics is attributed to the SVM algorithm, and the highest amount of ROC and PRC are related to the RF algorithm. Also, the lowest FP was related to the SVM algorithm. As a general rule, cross-validation epochs involve partitioning data into two complementary subsets. One of these sets will be used for training and fitting, and the other will be used for validation and testing. The results are averaged after numerous iterations of the validation using various subsets. The data are divided into K subsets for K-fold cross-validation, one of which is utilized for validation and the other K-1 for training. In this approach, each data fold is utilized exactly once for training and once for validation. As a result, we decide to base our final estimate on the averages of these K validation periods. Therefore, in this study, the 10-fold validation method was used, and the mean of the final results are presented. The obtained values of these metrics after testing ten times on the test dataset and averaging these ten values are shown in Table 4.

**Figure 2:**
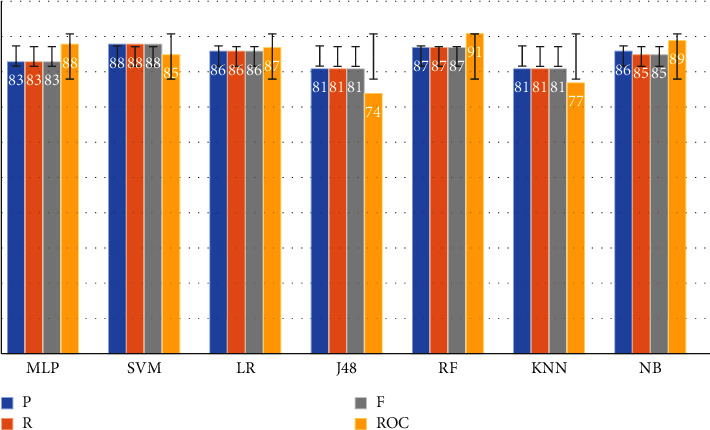
Comparison of the algorithm's metrics (P: Precision, R: Recall, F: F-measure, and ROC).

To provide a better comparison of the performance indicators of selected ML algorithms, such as precision, recall, F-measure, and ROC, a confusion matrix was used. After calculating the confusion matrix we concluded RF and SVM are the most effective approaches for the classification of CAD data.  Figure 2 depicts the ROC and other metrics for selected ML algorithms.

**Figure 3:**
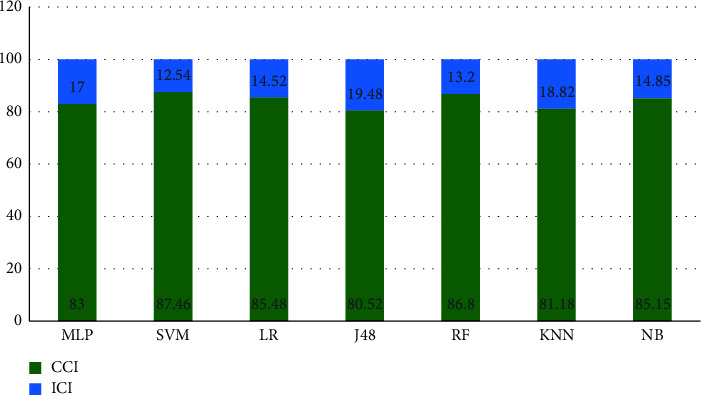
Correctly classified instances (CCI) and incorrectly classified instances (ICI) in the algorithms.

In order to accurately evaluate chosen ML algorithms, it is better to use more accurate metrics. Therefore, correctly classified instances (CCI) and incorrectly classified instances (ICI) were used in this study. Therefore, Figure 3 generally shows the amount of correctly detected and incorrectly detected samples based on different algorithms.

**Figure 4:**
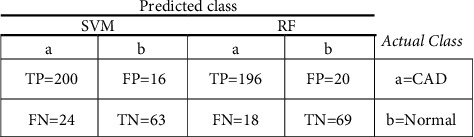
The confusion matrix of SVM and RF algorithms.

Two of the factors used to compare different ML algorithms are correctly classified instances (CCI) and incorrectly classified instances (ICI). In many studies, these metrics determine the performance of ML algorithms [36]. Based on the two indicators, CCI and ICI, SVM and RF algorithms had the highest efficiency among the run algorithms (Figure 2). Selected ML models employ the confusion matrix as a performance evaluation metric. The confusion matrix of the model evaluation on the test data is shown in Figure 4, which offers a clearer insight of the outcomes.

**Figure 5:**
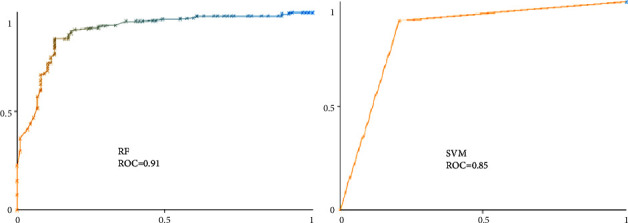
The ROC of the most efficient algorithms (SVM, RF).

ROC is an important index that includes a range of values for receiver operating characteristics (ROC). This term is used in signal detection to describe the tradeoff between hit rate and false alarm rate when the channel is noisy. ROC curves illustrate the performance of a classifier without regard to class distribution or error costs. They plot the true positive rate against the true negative rate. In Figure 5, we show the ROC diagram for both RF and SVM algorithms. Based on this diagram, it appears that these two algorithms have produced extraordinary results.

Each algorithm has specific parameters and settings. In this study, algorithms have been recorded to maintain the validity and reliability of the parameters and settings. In Table 5 the parameter value is presented. The selected algorithms were manually tuned with different parameters in order to improve their efficiency and minimize their errors. By varying these values, the speed of convergence, the learning steps, and the categories of feeding data to the models changed, resulting in the models reaching the most optimal state and the least error.

The algorithms were implemented and run using Weka v 3.5.9 software (The University of Waikato, Hamilton, New Zealand) and the results were presented to select the optimal algorithm (s) from the analysis in the form of graphs and comparison tables. In order to increase the efficiency of the selected algorithm (s), the properties of the algorithms, which are shown in Table 5, were manually adjusted and changed.

## 5. Discussion and Conclusion

Many studies have been conducted on CAD using ML algorithms, and significant results have been obtained in recent years. Many of these studies used paraclinical data to analyze CAD, some of which also used heart tissue imaging data. Using clinical examination criteria to design a CAD diagnosis model drastically reduces the cost and time of the diagnosis process. According to the findings of this study, when the CAD diagnostic model is used in a specific instance, the chance of its correctness is close to 90%. Therefore, the cardiologist can employ the ML models as an additional diagnostic tool to get a definitive determination.

ML algorithms have played a pivotal role in diagnosing coronary artery disease. It can become the basis for developing clinical decision support systems. SVM and RF algorithms had the highest efficiency and could diagnose CAD based on patient examination data. It is suggested that further studies be performed using these algorithms to diagnose coronary artery disease to obtain more accurate results. More precise disease prediction tools will be required to avoid coronary artery disease. Imaging methods, such as echo and esophageal echo, can play a decisive role in diagnosing CAD disease. Therefore, a combination of clinical data and cardiac imaging data and the use of newer artificial intelligence methods such as deep learning as a powerful tool can play an important role in predicting the occurrence of CAD.

Such ML models could be used with more extensive data, such as ECG features and other data, and examination data can help specialists detect coronary artery disease more correctly. Demographic data play a significant role in implementing ML-based models for disease prediction and diagnosis. According to the results, it can be claimed that variables such as age, sex, patient weight, BMI, and FH are effective in the initial diagnosis of CAD. So, it is suggested that these predictor variables for the diagnosis of coronary artery disease and patients' clinical examination variables should be used in studies based on ML. In other words, these variables, along with the variables of patients' clinical examinations, are somewhat indicative of the disease status and can help the physician make an accurate diagnosis. It seems that using a more comprehensive data set can increase the accuracy of patient prediction and diagnosis models. Because additional data can lead to greater pattern extraction and, consequently, a better understanding of the data's complexity in learning-based models; therefore, one of the limits of researching the limited amount of the dataset was employed because using big volume data will be one of the influential variables in achieving models with better accuracy and precision. Future research should take advantage of a more comprehensive data collection. Another drawback of the study was that the factors were limited to clinical examination and patient identifying information. Tests and ECGs are instrumental in detecting CAD; nonetheless, the variables were chosen based on the study's aims.

ML algorithms have played a pivotal role in diagnosing coronary artery disease. It can become the basis for developing clinical decision support systems. SVM and RF algorithms had the highest efficiency and could diagnose CAD based on patient examination data. It is suggested that further studies be performed using these algorithms to diagnose coronary artery disease to obtain more accurate results. More precise disease prediction tools will be required to avoid coronary artery disease. Imaging methods such as echo and esophageal echo can play a decisive role in diagnosing CAD disease. Therefore, a combination of clinical and cardiac imaging data and the use of newer artificial intelligence methods such as deep learning as a powerful tool can play an important role in predicting the occurrence of CAD.

## Figures and Tables

**Table 1:** Studies evaluating machine learning algorithms used for CAD detection.

Authors, year, country	Aim of study	Data and features	Sample size	ML method and algorithms	Performance			Validation technique	Detail
Abdar et al. 2019, Poland [16]	Accurate diagnosis	Alizadeh dataset: demographic; symptom, examination, ECG, laboratory, echo	303	C-SVC, NU SVC, linear SVM	F1score = 91.51 Acc = 93.08		10-fold		One hot encoding, genetic algorithm, genetic optimizer

Gupta et al. 2019, Canada [17]	Estimating the risk of CAD	Z-Alizadeh Sani (demographic, health history, medical procedure features)	303	BN (Bayesian network)	AUC = (0.93 + 0.04)		10-fold		LR, SVM, ANN graphical reasoning introduces

Joloudari et al. 2020, Iran [18]	CAD diagnosis	Z-Alizadeh Sani dataset	303	DT (Decision tree)	AUC = 91.47	10-fold			RTS (Random tree), SVM, DT

Tama et al. 2020, S. Korea [19]	Detection CHD	5 dataset (Z-Alizadeh Sani, statlog, cleveland, Hungarian)	303	Two-tier ensemble (GBM, GXboost, RF)	Proposed AUC > other ensemble and individual models	10-fold			Random forest (RF), gradient boosting. Correlation-based feature

Iong et al. 2021, Taiwan. [20]	Early prediction of CAD	7 feature (demographic and medical history)	NM	SVM with pooling layer	SVM	NM			SVN NB

Chen et al. 2020, China [21]	Detection of CAD	1163 variables (morphological)		Polynomial SVM with grid search optimization	Acc = 100%	10-fold			LR, DT, LDA, KNN, ANN.SVM

Zhang et al. 2020, China [22]	Detection of CAD	Holter monitoring, echocardiography (ECHO), and biomarker levels (BIO)	62	Holter model	Sen = 96.67% Spe = 96.67% Acc = 96.64%	5-fold			Random forest, and SVM. Bioexamination reach the best result.

Ricciardi et al. 2020, Italy [23]	Prediction of CAD	22 features (laboratory and medical history)	10,265	LDA and PCA	Acc = 84.5 and 86.0 Spe > 97% Sen > 66%	10-fold			PCA and LDA for feature extraction PostgreSQL, a DBMS

Pattarabanjird et al., 2020, USA [24]	Prediction of CAD severity	Demographics and laboratory	481	NN (ID3 rs11574)	AUC = 72% to 84%	NM			Crf, ID3

**Table 2:** Specifications of the dataset features used in the study.

Feature name	Type of feature	Missing	
Age	Numeric	0	M = 59 ± 10
Weight	Numeric	0	73.83 ± 12
Length	Numeric	0	165 ± 9.33
Sex	Nominal	0	Male = 176, female = 127
BMI (body mass index)	Numeric	0	27.25 ± 4.01
Diabetes mellitus (DM)	Nominal	0	Yes, no
Hypertension (HTN)	Nominal	0	Yes, no
Current smoker	Nominal	0	Yes, no
Exsmoker	Nominal	0	Yes, no
Family history (FH)	Nominal	0	Yes, no
Obesity	Nominal	0	Yes = 211, no = 92
Chronic renal failure (CRF)	Nominal	0	Yes = 6, no = 297
Cardiovascular diseases (CVA)	Nominal	0	Yes = 5, no = 298
Airway diseases	Nominal	0	Yes = 11, no = 292
Thyroid diseases	Nominal	0	Yes = 7, no = 296
Congestive heart failure (CHF)	Nominal	0	Yes = 1, no = 302
Dyslipidemia (DLP)	Nominal	0	Yes = 112, no = 191
Edema	Nominal	0	Yes, no
Weak peripheral pulse	Nominal	0	Yes = 5, no = 298
Systolic murmur	Nominal	0	Yes = 41, no = 262
Diastolic murmur	Nominal	0	Yes = 9, no = 294
Typical chest pain	Nominal	0	Yes, no
Dyspnea	Nominal	0	Yes = 134, no = 169
Atypical	Nominal	0	Yes = 93, no = 210
Nonanginal	Nominal	0	Yes = 16, no = 287
Catheterization (cath)	Nominal	0	CAD = 216, normal = 87

**Table 3:** The ratio of dividing the original dataset into sets for training, testing, and validation.

	Training set	Testing set	Validation set	Total
Percentage (%)	80%	16%	4%	100%
No. of paired samples	242	48	13	303

**Table 4:** Performance comparison of seven ML algorithms for diagnosis CAD using different evaluation metrics.

Algorithms	TP	FP	Precision	Recall	*F*-measure	MCC	ROC	PRC
MLP	0.83	0.25	0.83	0.83	0.83	0.59	0.88	0.88
SVM	0.88	0.17	0.88	0.88	0.88	0.70	0.85	0.83
LR	0.86	0.21	0.86	0.86	0.86	0.65	0.87	0.86
J48	0.80	0.28	0.81	0.81	0.81	0.52	0.74	0.74
RF	0.87	0.22	0.87	0.87	0.87	0.67	0.91	0.91
KNN	0.81	0.28	0.81	0.81	0.81	0.54	0.77	0.77
NB	0.85	0.18	0.86	0.85	0.85	0.65	0.89	0.88

**Table 5:** The specifications and settings of algorithms including parameters.

Algorithm	Setting
MLP	Batch size = 100, learning rate = 0.3, momentum = 0.2, number of decimal places = 2
		SVM	Batch size = 100, the polynomial kernel: *K* (*x*, *y*) = <*x*, *y*>^*p* or *K* (*x*, *y*) = (<*x*, *y* >+ 1) ^*p*, random seed = 1, tolerance parameter = 0.001 number of decimal places = 2
LR	Batch size = 100, max lets = −1, ridge = 1.0*E*−8, number of decimal places = 4		
		J48	Batch size = 100, confidence factor = 0.25, min num obj = 2, num folds = 3, seed = 1, number of decimal places = 2
RF	Batch size = 100, number of iterations = 100, seed = 1, number of decimal places = 2		
		KNN	Batch size = 100, number of decimal places = 2
NB	Batch size = 100, number of decimal places = 2		

## Data Availability

The data used to support the findings of this study are available publicly at https://archive.ics.uci.edu/ml/datasets/Z-Alizadeh+Sani.
